# Simulation of transport processes and chemical reactions in building materials

**DOI:** 10.1186/1758-2946-5-S1-P13

**Published:** 2013-03-22

**Authors:** Carina Jehn, Frank Schmidt-Döhl

**Affiliations:** 1 Institute of materials, physics and chemistry of buildings, Hamburg University of Technology, Hamburg, Germany

## 

Usually the prediction of the lifetime of building materials is based on costly and time-consuming laboratory experiments. Numerical simulation of transport processes and the resulting chemical reactions in building materials is a versatile tool to estimate durability and corrosion processes of structures. Several studies exist on the characterization of reactions in porous building materials and the resulting deterioration [1].

Chemical reactions between corrosive media and mineral building materials take place on the material surface as well as in the pore volume. Thus the program TransReac combines two main modules: chemical reaction involving thermodynamics and kinetics and the transport of species into pores [1,2].

Using thermodynamic parameters of involved species and experimental determinated transport characteristics of the examined building materials, reactions depending on time and position were simulated. Gibbs energy, density and other thermodynamic parameters, e.g. heat capacity can be found in [3,4]. This database is proved successful in building material research.

Different kind of chemical attack to mineral building material were simulated and verified by experimental data, e.g. corrosion caused by sulphate or ammonium solutions, acid and debonding of gypsum plaster [1,2]. The paper is concentrated on the dissolution of silica from aggregates in alkaline concrete pore solution at various temperatures, in regard to the simulation of the initial phase of alkali-silica-reaction of concrete.
